# Occurrence of inadequate ACL healing after Dynamic Intraligamentary Stabilization and functional outcome—a multicentre case series

**DOI:** 10.1007/s00590-021-03096-9

**Published:** 2021-08-24

**Authors:** Monika Senftl, Daniel Petek, Matthias Jacobi, Alex Schallberger, Jonathan Spycher, Anna Stock, Rolf Hess, Moritz Tannast

**Affiliations:** 1 Department of Orthopaedic Surgery, Fribourg Hospital, Villars-sur-Glâne, Switzerland; 2 Orthopaedics Rosenberg, St Gallen, Switzerland; 3 Department of Orthopaedic Surgery, Interlaken Hospital, Unterseen, Switzerland; 4 Department of Orthopaedic Surgery, Thun Hospital, Thun, Switzerland; 5 University of Fribourg (UNIFR), HFR Cantonal Hospital, Ch. des Pensionnats 2-6, 1700 Fribourg, Switzerland

**Keywords:** ACL tears, ACL self-healing capacities, Dynamic Intraligamentary Stabilization

## Abstract

**Introduction:**

Dynamic Intraligamentary Stabilization (DIS) is a technique for preservation, anatomical repair and stabilization of a freshly injured anterior cruciate ligament (ACL). The main purpose of this study was to evaluate the short-term re-operation rate when compared to traditional autograft reconstruction.

**Methods:**

Four, from the developer independent, centres enrolled patients that underwent ACL repair by DIS, according to the specific indications given by MRI imaging at a minimum follow-up of 12 months. The re-operation rate was recorded as primary outcome. Secondary outcome measures were the postoperative antero-posterior knee laxity (using a portable Rolimeter®), as well as the Tegner, Lysholm and IKDC Scores.

**Results:**

A total of 105 patients were investigated with a median follow-up of 21 months. Thirteen patients were lost to follow-up. Of the remaining 92 patients 15 (16.3%) had insufficient functional stability and required subsequent ACL reconstruction. These patients were excluded from further analysis, leaving 77 consecutive patients for a 12 months follow-up. The median age at time of surgery was 30 years for that group. At time of follow-up a median antero-posterior translation difference of 2 mm was measured. None of these patients reported subjective insufficiency (giving way), but in 14 patients (18.2%), the difference of antero-posterior translation was more than 3 mm. We found a median Tegner Score of 5.5, a median Lysholm Score of 95.0 and a median IKDC Score of 89.4.

**Conclusion:**

The main finding of this multicentre study is a relevant re-operation rate of 16.3%. Another 18.2% showed objective antero-posterior laxity (≥ 3 mm) during testing raising the suspicion of postoperative non-healing. The failure rate of DIS in this study is higher than for reconstruction with an autologous tendon graft. However, our successfully treated patients had a good clinical and functional outcome based on antero-posterior knee laxity and clinical scores, comparable to patients treated by autograft reconstruction.

## Introduction

Anterior cruciate ligament (ACL) injuries in the knee joint are common and their incidence continues to increase. The annual reported incidence in the USA alone is approximately 1 in 3500 people [1]. In order to restore function in patients with significant knee instability, surgery is often indicated as high-grade partial and complete ACL tears rarely heal. The rate of anterior cruciate reconstruction in the USA is constantly raising particularly in females as well as in patients younger than 20 years and those older than 40 years of age [2]. Initially described as preventing the occurrence of osteoarthritis of the knee, the ACL reconstructions have shown long-term high rates of radiographic osteoarthritic changes with an almost three times increase in prevalence [3]. Standard predictors for the development of knee osteoarthritis after ACL reconstruction include prior meniscectomy (medial or lateral), medial meniscectomy at the time of reconstruction, and elevated body mass index [4]. Conservative treatment after ACL rupture does not give satisfactory results in the long term either, and a delay to reconstruction of more than 12 months is associated with increased meniscal or chondral injuries [5]. Current research focuses on reconstruction techniques, biomechanical evaluation and anatomy. Recently, new devices and surgical techniques that promote a self-healing of the ACL tear have been developed and brought on the orthopaedic market with the hypothesis of a better clinical and radiological outcome [6–8].

### Anatomy and physiology

The anterior cruciate ligament (ACL) plays a crucial role in stability of the knee and is the primary restraint to anterior translation of the tibia at all degrees of flexion [9, 10]. Due to its intra-articular but extrasynovial location with immediate contact with a synovial fold containing vascular and neural elements, the ACL plays an additional important proprioceptive function [11]. Anatomic studies have shown that the ACL originates from the posterior aspect of the medial surface of the lateral femoral condyle and inserts onto the tibia between the intercondylar eminences [12]. The average length is 38 mm and the average width is 11 mm [10, 13]*.* It is composed of two bundles and its many fascicular subunits are selectively recruited during tensile loading making it a unique structure difficult to reconstruct especially because of all subtleties of knee motion [14].

### Treatment of ACL tears

ACL tears are among the most common injuries during sport activities. Poorly treated, they may prevent proper knee kinematics and lead to functional impairment [15, 16].

The poor rate of primary healing observed after ACL rupture is believed to be multifactorial in nature; including an unfavourable, intra-articular biologic environment, a compromised blood supply of the ligament and a persistent post-traumatic instability preventing the torn ligament from healing [17]. Primary repair of the torn ACL has been widely abandoned after arthroscopic ACL reconstruction became the standard of care in the early 1990s and superseded the results of primary repair.

Despite advances in surgical techniques, ACL reconstruction is not a universally successful procedure and may present non-negligible rates of recurrent laxity [18]. The reconstruction of the unique anatomy of the ACL in all its subtleties is a difficult issue, due to the interdigitated construction of the different bundles.

The optimal surgical technique, the graft choice and the timing of surgery for the reconstruction of the ACL are controversially discussed in contemporary orthopaedic literature. Among other reasons, the unsatisfying functional outcome in ACL reconstruction could be explained by a decreased proprioception [11] due to removal of the native ACL tissue containing sensory nerve fibres and by replacing it with a non-vital graft [6]*.*

## Dynamic intraligamentary stabilization

To address the loss of proprioceptive inputs and to preserve the native ACL fibres, recent studies have focused on new strategies, which support ACL’s potential of biological self-healing. In order to overcome two potential inhibitors of successful ACL healing, (compromised blood supply and excessive tension at the scar tissue site), a novel technique with a dynamic augmentation and stabilization of primary ACL repair called Dynamic Intraligamentary Stabilization (DIS), was developed.

In preliminary studies [6–8, 19], the freshly injured ACL was preserved, anatomically reduced and intraarticularly stabilized instead of being removed and replaced. A supportive spring-screw system (Ligamys™, Mathys Ltd Bettlach, Switzerland) was implanted to shield cyclic loads acting upon the ACL, thus helping to maintain adequate antero-posterior knee stability during the healing phase [6]*.* The healing process of the torn ligament leads to a living, proprioceptive active ligamentous structure that aims to restore the stability and kinematics within the knee joint [20].

Eggli et al. [20] showed that an anatomical reposition with DIS and a stabilization of the stumps with a dynamic spring system leads to a clinically stable healing of the torn ACL in the large majority of patients. The first results showed promising functional outcomes and most patients exhibited almost normal knee function and reported excellent satisfaction rates. These findings support this new technique in ACL treatment increasing the chance for self-healing of the ACL compared to conservative treatment or graft replacement.

The purposes of this multicentre case series were to analyse the stability of the knee and to evaluate the functional outcomes after ACL repair by DIS as well as to compare these results with those of the current literature. Also it has been hypothesized that the revision surgical rates and clinical and functional outcomes would be similar to the results of the developer group.

## Material and methods

This study analysed a retrospective, consecutively documented multiple-centre case series using a DIS device and the primary endpoint was insufficient stability leading to a revision surgery. All patient data have been fully anonymized and collected according to institutional board recommendations.

Four, from the developer independent centres, enrolled patients that underwent arthroscopic ACL repair by DIS with a minimum follow-up of 12 months. In centre 1 and 2, one single surgeon undertook the procedure, whereas in centre 3 and 4, up to 4 different senior surgeons did the surgical procedure. All surgeons have been trained for the DIS procedure prior to start and emphasis has been given on proper indication according to MRI studies and surgical delay from the accident.

Inclusion criteria were an acute ACL rupture confirmed by MRI and possible to treat within 21 days, regular participation in sports and patient not eligible for conservative treatment. Conservative treatment was recommended if the following criteria were fulfilled: no more than a 3 mm difference in AP translation (Rolimeter®) when compared with the uninjured contralateral side; no participation in pivoting sports and no meniscal lesions.

Exclusion criteria were as follow: concomitant ligamentory or injuries, concomitant fractures, previous surgery on the injured knee, prior restriction in sports activity, bilateral injuries, multiple injured patients and obese patients with a BMI greater than or equal to 30.

### Operative technique

The surgical procedures were performed by 8 experienced senior orthopaedic surgeons who had attended a training course to familiarize with the method and the technique involved.

All procedures were performed under either spinal or general anaesthesia. Patients were placed in supine position with the knee positioned in a dynamic leg holder. A tourniquet was used in all cases.

**Fig. 1:**
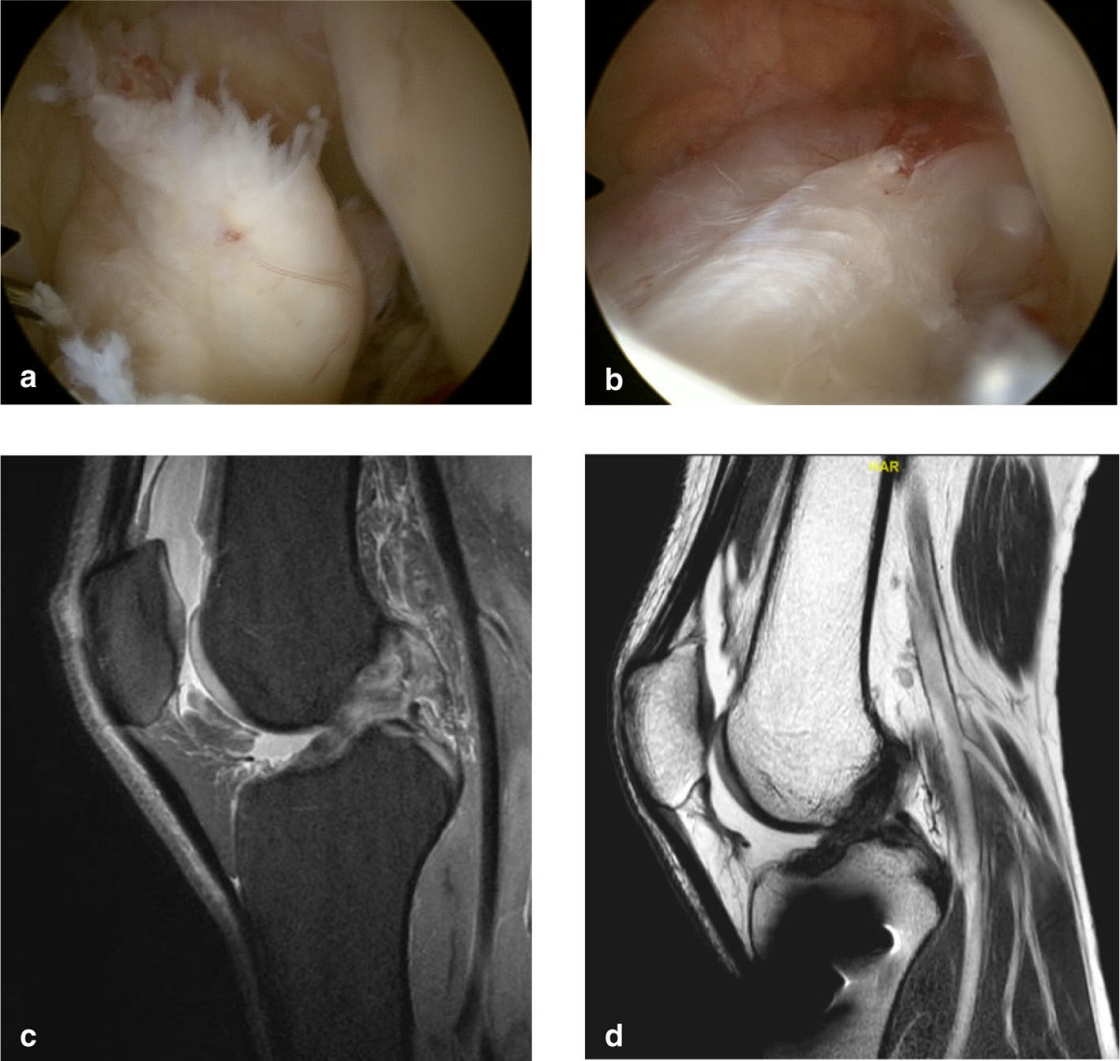
**a**-**d**, **a** preoperative view of the ACL tear, **b** preoperative view after DIS implantation, **c** preoperative MRI ACL tear, **d** postoperative MRI view with healed ACL (6 months)

The operative technique consisted of individualizing the torn ACL bundles, which were approximated and guided to the femoral footprint by transosseous resorbable sutures. After performing microfractures at the femoral footprint, the knee joint was guided in a posterior drawer position with a strong polyethylene wire, which was passed posterior to the tibial footprint and through the anatomical femoral footprint preserving the original insertions with the blood supply for the ACL (Fig. 1). This wire was brought under tension (60–80 N, depending on weight and gender) and anchored by a spring-screw implant (Ligamys™, Mathys Ltd Bettlach, Switzerland), which was placed on the antero-medial aspect of the tibia. Thus, the knee is held in a constant posterior drawer translation. The spring mechanism allowed a dynamic excursion of 8 mm, ensuring a continuous tension of the wire at every degree of flexion [20, 21].

### Postoperative rehabilitation

The first three days, the knee was kept in extension to enable adhesion of the ACL stumps. The leg was then loaded with 15 kg of weight for 3 weeks and progressively mobilized in flexion and extension. Beginning at 4 weeks postoperative, full weight bearing was allowed and reinforcement training was initiated using closed chain knee exercises followed by proprioceptive training and balancing exercises. Unlimited training was allowed after 10 weeks and pivoting sports after 6 months. If a meniscal suture was done concomitantly, the postoperative rehabilitation was adapted accordingly.

### Post-operative evaluation and outcome measure

The re-operation rate was recorded. Secondary outcome measures were antero-posterior knee laxity and clinical functional scores.

The knee laxity was assessed by measuring the anterior tibial translation at 30 degrees of flexion using a portable arthrometer (Rolimeter®, Neubeuern, Germany) and comparing the obtained value with the contralateral knee. The value used was that from the last available follow-up.

The clinical outcome was evaluated by using the following scores: Lysholm and International Knee Documentation Committee (IKDC) score, self-reported Tegner activity scale [22] and visual analogue scale for pain and patient satisfaction. Patient’s subjective perceived impairment was analysed on a visual analogue scale (VAS) (0 = completely dissatisfied, 10 = completely satisfied). The activity level was determined using the self-reported Tegner score. The pre-injury scores were assessed retrospectively.

The clinical examination consisted of an assessment of effusion, range of motion (ROM) and testing of rotational stability by a pivot shift test.

All patients were informed that their treatment and follow-up data would be recorded in a scientific database for evidence generation, for which they gave their voluntary written general informed consent.

### Statistical analysis

Data were exported to SPSS version 22.0 software (SPSS Incorporated, Chicago, Illinois) for statistical analyses. All statistical comparisons were conducted using 95% confidence intervals where a p value of less than 0.05 was considered statistically significant. To express the variability and distribution of the underlying data, the median values and their range were calculated and reported.

Univariate analysis was performed by means of the Chi-squared test for binary, logistic regression for numerical variables, and Wilcoxon rank-sum test for Tegner score.

## Results

### Characterization of the study population

Between December 2013 and January 2015, a total of 105 patients were treated with a DIS in 4 different centres. The median age at time of surgery was 28 years (range 14–57 years) (mean 30.8 years). The patients were included for clinical follow-up with a median follow-up of 21 months (range 12–26 months). Thirteen patients (12%) were lost to follow-up or went abroad.

### Reported postoperative data

Of the remaining 92 patients 15 (16.3%) had insufficient stability (painful giving ways or knee distortion) during pivoting activities. These patients had a significant antero-posterior translation and required subsequent ACL reconstruction within the period of 26 months. These patients were excluded from further analysis. This left 77 consecutive patients for follow-up (male–female-ratio 38:39) with a median age at time of surgery of 30 years (range 14–57 years) (mean 32.8 years).

This population was treated as follows: 17 (22.1%) were treated in centre 1, 15 (19.5%) in centre 2 and 18 in centre 3 (23.4%) and 27 (35.1%) in centre 4 (Table 1).

**Table 1:** Patient data and demographics

Centre	Number of patients	Sex-ratio: male–female	Age median (range years)	Side ratio: dominant–nondominant
1	17	10:7	25 (16–50)	10:7
2	15	8:7	25 (14–57)	10:5
3	18	6:12	38 (17–54)	10:8
4	27	14:13	30 (16–53)	17:10

Overall, 25 (32.5%) patients showed additional meniscal lesions, which were surgically addressed by suture when located in the red-white zone.

**Fig. 2:**
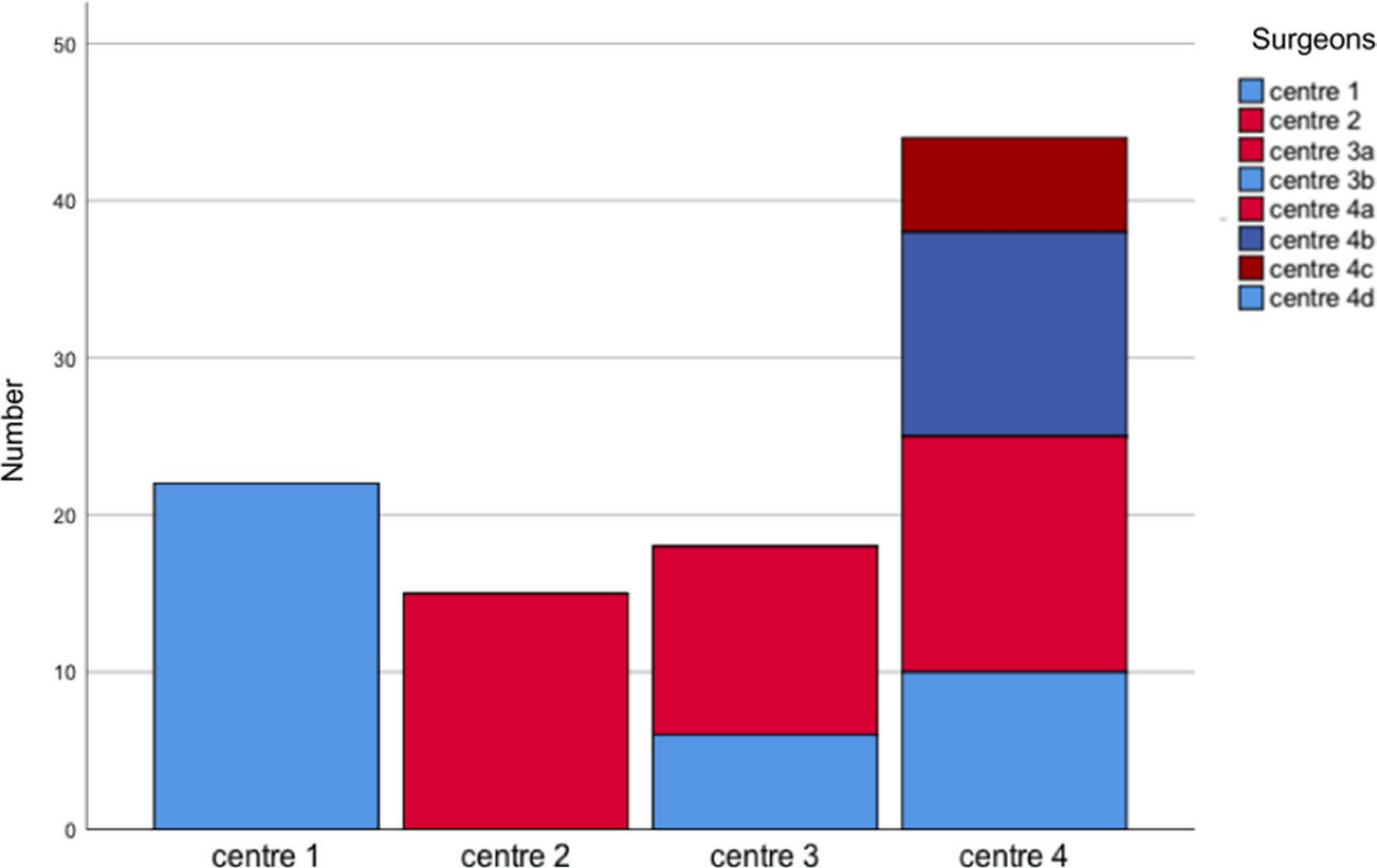
Patient distribution according to treatment centre and surgeon

The age distribution at time of surgery shows comparable values except for centre 3 where the age group was higher (Fig. 2).

### Patient outcome measures and clinical evaluation

**Fig. 3:**
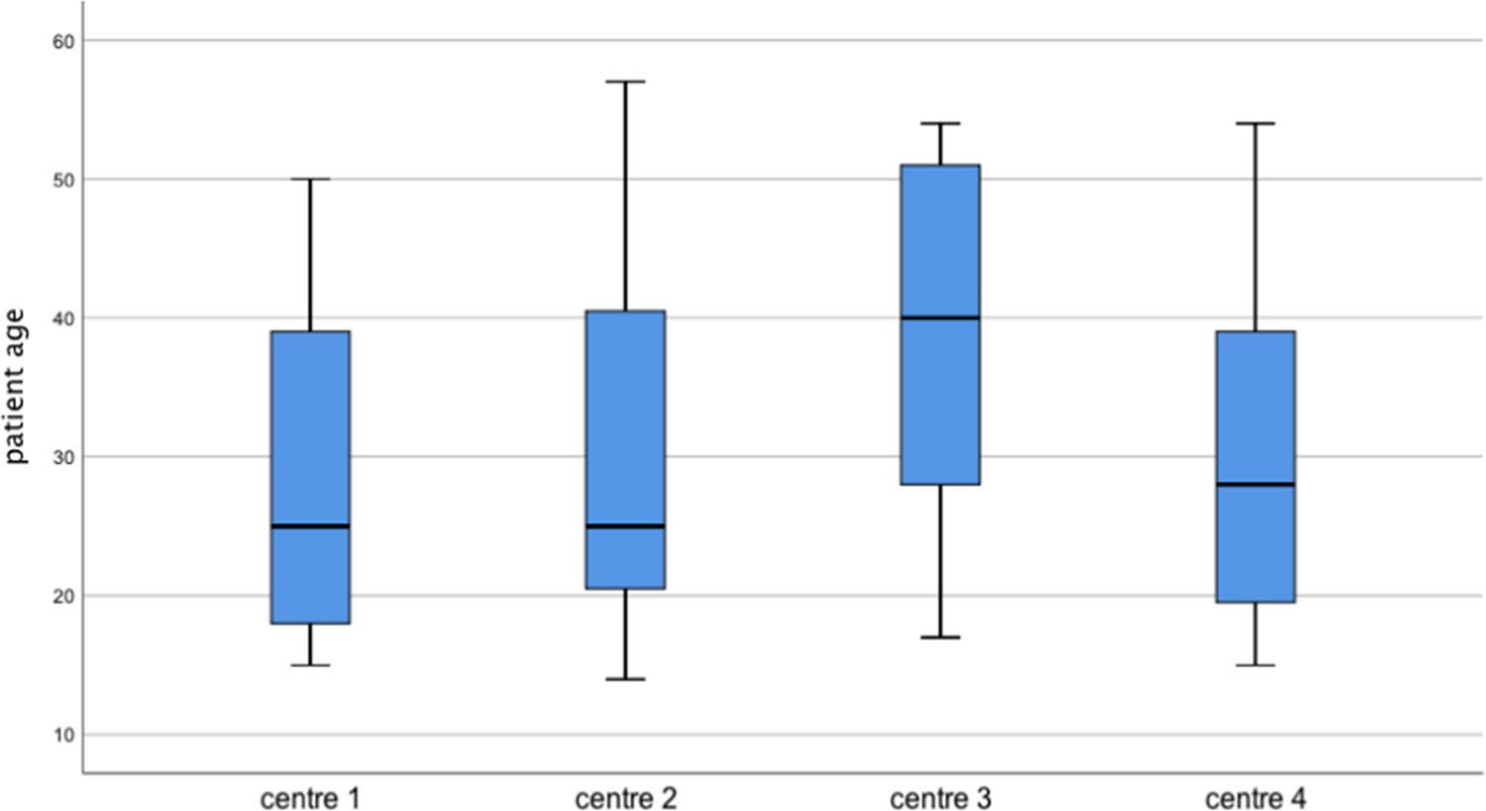
Age distribution at time of surgery

At 12 months follow-up, we found a median difference in antero-posterior translation (Rolimeter®, side-to-side difference) of 2 mm (range 0–6 mm). Detailed analysis revealed that 26 patients (33.7%) had ≤ 2 mm difference. An antero-posterior translation difference between 2 and 3 mm was observed in 37 patients (48.1%) and more than 3 mm in 14 patients (18.2%) (Fig. 3).

**Fig. 4:**
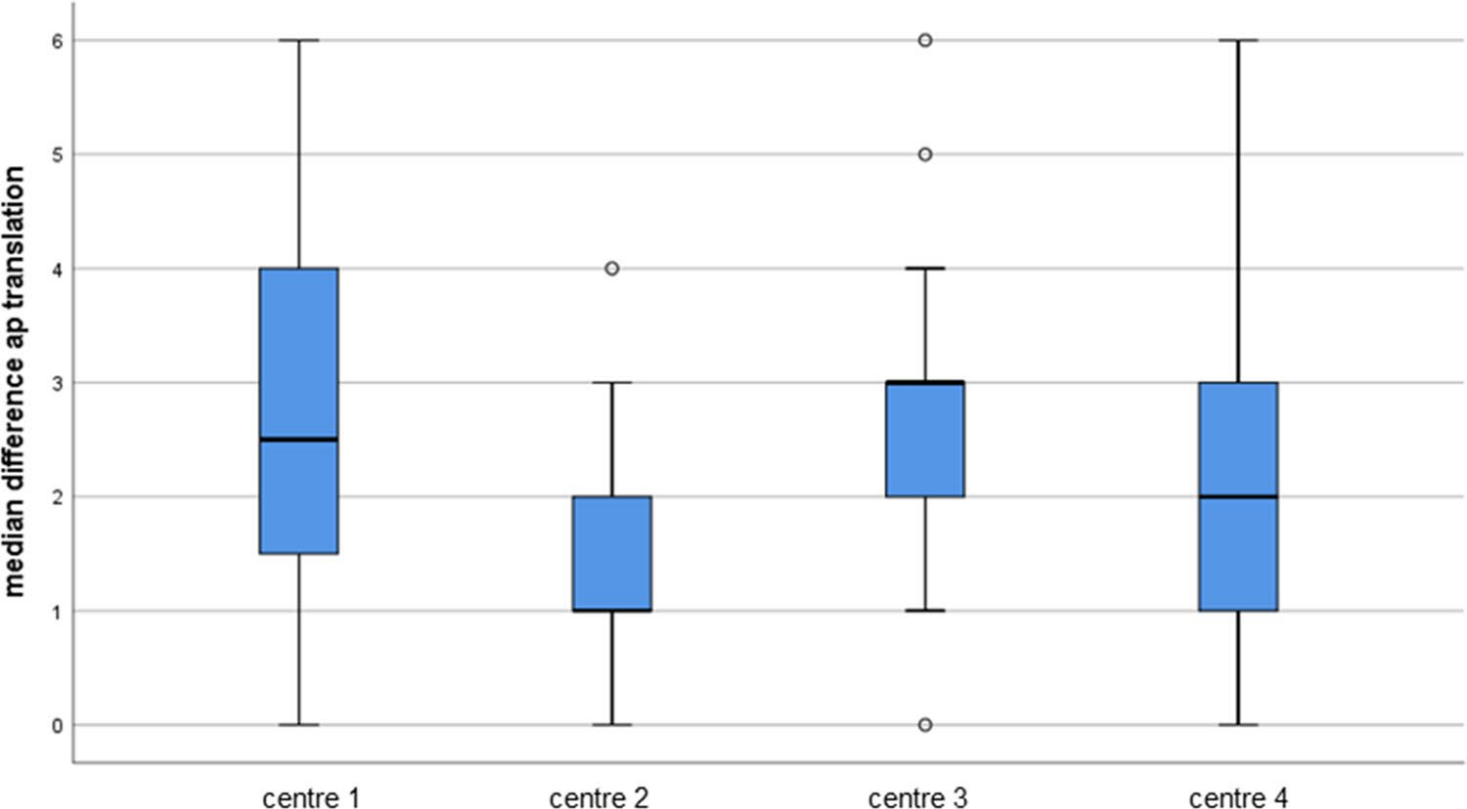
AP-translation difference according to treatment centre

Overall, 45 patients (58.4%) had less than 3 mm side-to-side difference (Fig. 4).

The functional outcome measures reported to the patient revealed a median Lysholm score of 95 (range 53–100) and a median IKDC score of 89.4% (range 55–100%).

The median IKDC grading was B (nearly normal/good) in 20 patients (25.9%); A (excellent) in 37 patients (48.1%), C (abnormal/fair) in patients 16 (20.8%) and D (poor) in 4 patients (5.2%).

The median Tegner activity scale prior to injury, assessed through patients' medical history, was 6 (range 5–10) and remained postoperatively at the same pre-injury level (5.5; range 2–10). However, only 40 patients (51.9%) returned to their previous level of activity.

When a combined success definition (AP-translation difference ≤ 3 mm, Lysholm score > 84 points, IKDC score > 84%) was applied, 61.0% (47 patients) fulfilled all 3 criteria at last follow-up.

Most patients experienced almost no pain with a median VAS scale (0–10) of 1 (range 0–4) and patient satisfaction was fully achieved with a median satisfaction score of 9 points (range 3–10).

Clinical examination revealed two patients (2.6%) with joint effusion. Range of motion (ROM)-testing (flexion–extension) showed postoperatively on the operated side a median ROM of 140–0–0 degrees and on the contralateral side a median ROM of 145–0–3 degrees. Compared to the contralateral healthy limb, 16 (20.7%) patients demonstrated a flexion deficit of 5–10° and three (3.9%) patients of 15° or more. Fifteen (19.5%) patients had an extension deficit of ≤ 5 degrees. Five (6.5%) patients presented an extension deficit of more than five degrees.

### Objective and subjective instability

Objective instability has been defined as a positive pivot shift or delta-AP translation > 3 mm. Subjective instability has been defined as giving ways in torsional exercises.

Thirteen (16.9%) patients had a positive pivot shift testing and 14 patients (18.2%) presented an antero-posterior translation of more than 3 mm. Only 2 patients were positive in both testing. There were no reported subjective insufficiencies of the ACL (giving way).

The patients who needed a re-operation, presented subjectively giving ways and objectively an antero-posterior translation difference of ≥ 3 mm.

**Fig. 5:**
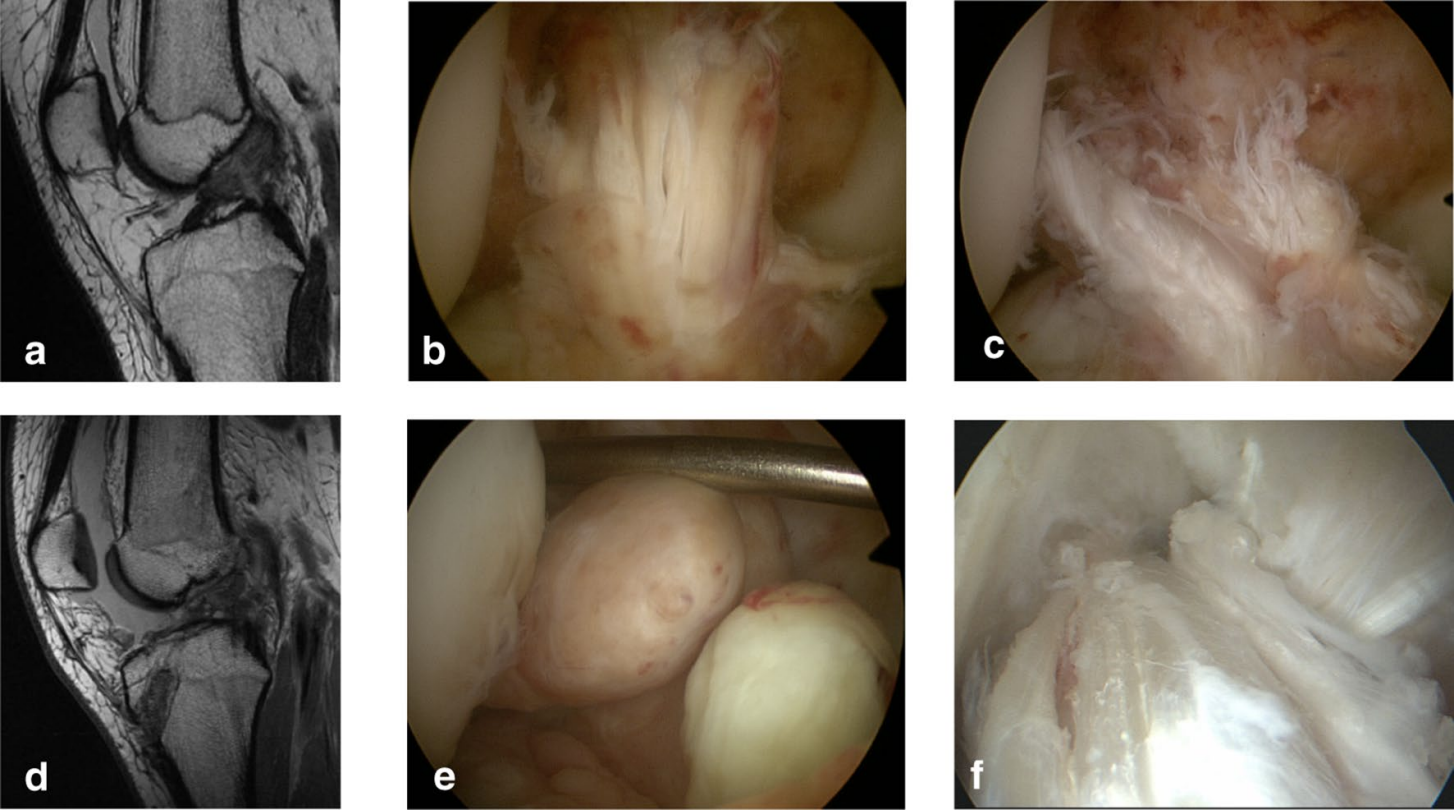
**a** MRI view of ACL rupture,** b** arthroscopic view of the ACL tear,** c** arthroscopic view DIS repair,** d** MRI view of the non-healed ACL repair,** e** arthroscopic view of the non-healed ACL,** f** arthroscopic view of the notch after autograft ACL reconstruction

The failure occurred within 14.6 months postoperatively and 9 cases (60%) had implant removal before. All re-ruptures were submitted to reconstruction by means of an autologous tendon graft (Fig. 5).

**Fig. 6:**
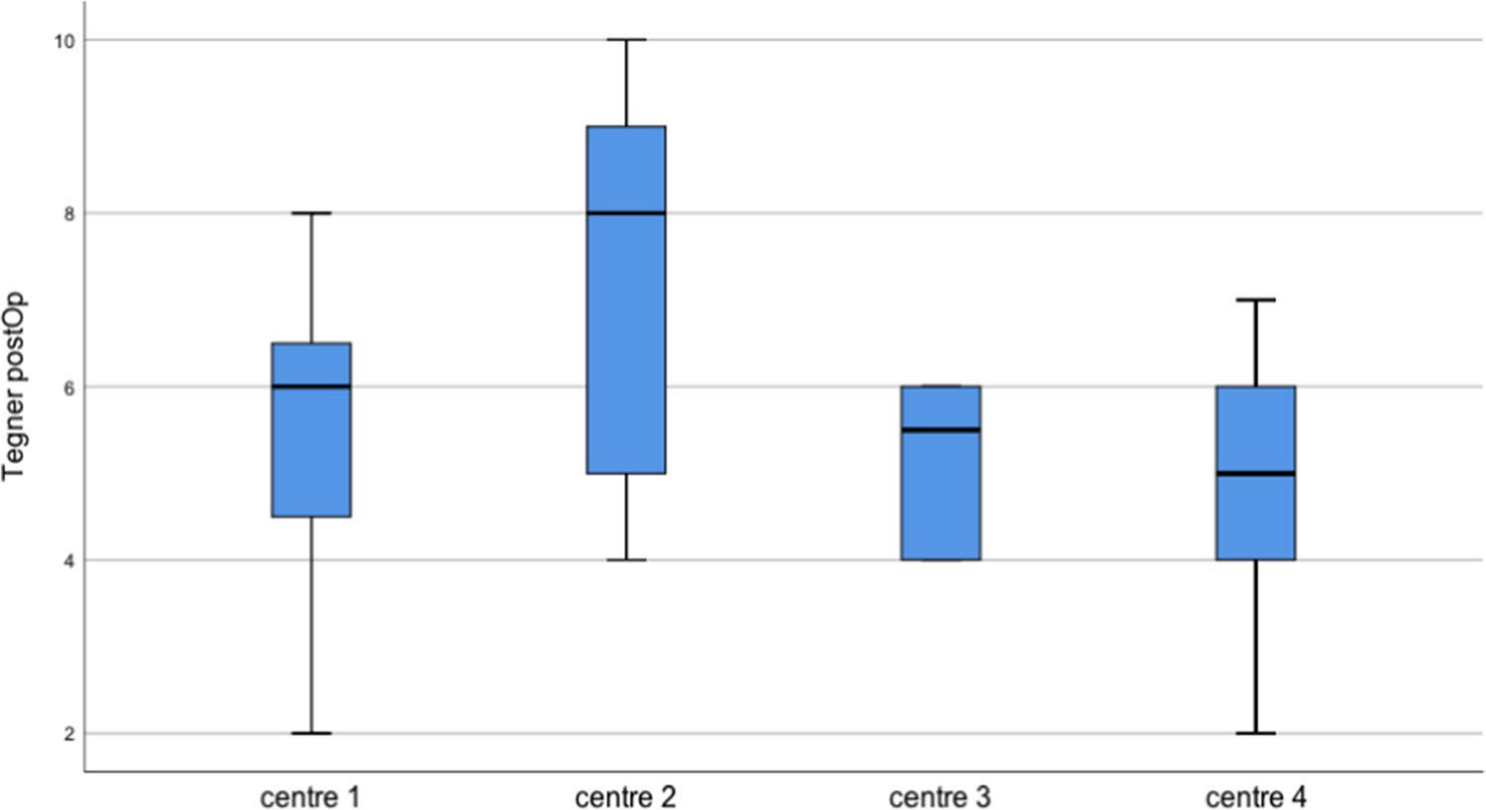
Postoperative Tegner activity scale according to treatment centre

**Fig. 7:**
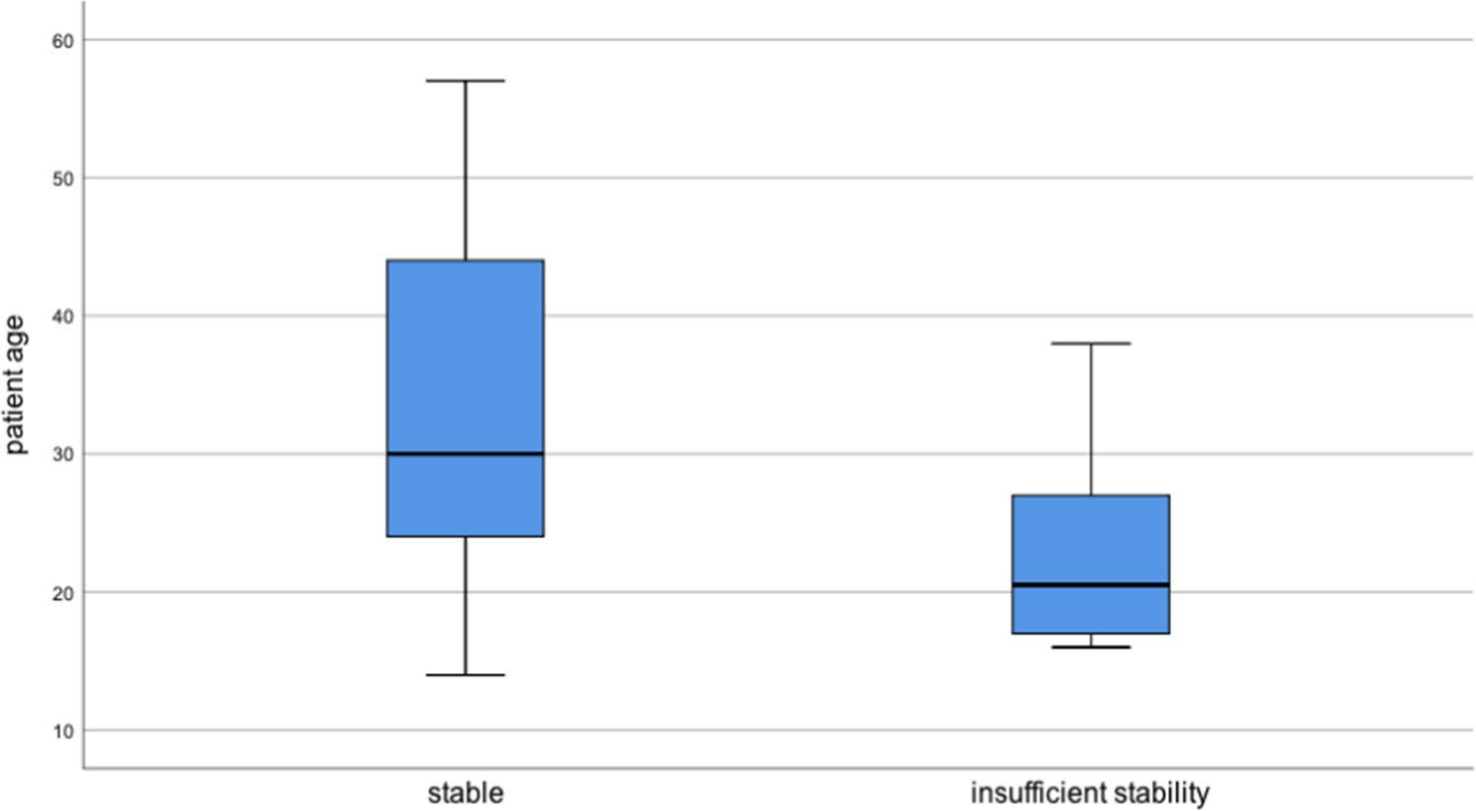
Median patient age compared to stability

The median age of patients with insufficient stability was 19 years (mean 21.1 years), whereas the median age of stable repairs was 30 years (mean 33.2 years) (Figs. 6 and 7).

### Reoperation rate

In total, 15 (16.3%) patients suffered from subjective and objective instability and required a standard ACL reconstruction. Forty-eight patients (62.3%) underwent a second procedure for implant removal after ACL healing. Not all patients required tibial hardware removal but it was offered to all patients after 6 months to restore a native knee.

## Discussion

The primary purpose of this developer independent study was to determine the incidence of revision surgery after a DIS procedure which was relevant revision reaching 16.3% by the end of this study. All re-ruptures were submitted to reconstruction by means of an autologous tendon graft.

Forty-eight patients (62.3%) underwent a second procedure for implant removal after ACL healing.

Kohl et al. [23] described their experience with the DIS technique in a single centre series of 50 patients and reported a 18% re-operation rate at two years. In addition, 30 patients (60%) required removal of the tibial screw.

Büchler et al. [24] mentioned 3 re-ruptures in their 45-patient cohort at one-year follow-up (6.7% re-rupture rate). A revision rate of 3.95% was reported by Henle et al. [20] in their case series of 278 patients and the implant was removed in 67 (24.1%) patients after DIS over a period of three years. In addition, three patients of the same cohort presented with a subjective giving way as a sign of insufficient healing but did not undergo revision surgery.

Five years after an ACL reconstruction by means of an autologous patellar or hamstring tendon graft, Salmon et al. [25] found a 6.4% re-rupture rate in 612 patients. A systematic review by Crawford et al. [26] showed a 6.2% graft rupture rate after ACL reconstruction.

The failure rate of DIS in the present study is higher than that reported by the developer and higher than for reconstruction with an autologous tendon graft. Interestingly, except for one patient, all re-ruptures tend to happen in a population ages lower than median value. We found a potential bias of insufficiency rate by age group: The lowest failure rate of 5.3% was at centre 3 Interlaken with a mean age of 36.7 years (median 27). In centre 4, we found the largest rate with 25.6% of failures. There, the mean age was 29.9 years (median 21). The centre 1 showed 14.3% of failures with a mean age of 28 years (median 23) and the centre 2 showed 17.6% of failures with a mean age of 30.4 years (re-rupture median 22).

Recently, published data focused on factors influencing the success of DIS. Henle et al. [27] showed in a ROC analysis an increased risk for revision ACL surgeries for younger patients and identified the age of 24 years as the cut-off separating high- and low-risk groups. Krismer et al. [28] showed that factors acting as determinants of poor outcome were mid-substance ACL tears and high pre-injury sports activity level.

Henle et al. [27] observed an incidence of revision ACL after DIS of 7.9% over 2.5 years of follow-up and found as well that young age and high baseline activity level were associated with an increased risk of ACL revision after DIS.

Still, ACL repair eliminates the drawback of reconstruction such the morbidity associated with the harvesting of grafts. The developer group suggests that a revision situation after DIS is advantageous since all grafts are still available for reconstruction. However, if the bulky tibial implant of DIS has not yet been removed, it may require a two-stage revision surgery with a 6-month interval between removal of the monoblock component along with bone grafting of the tunnel and ACL reconstruction by means of an autologous tendon graft.

It has to be discussed if a second intervention for implant removal should be mandatory or if it can be seen more as an option for the patient. Many patients requested removal of the screw even without clinical symptoms due to the implant. In this study, the implant was removed postoperatively in 62.3% of patients, restoring an almost native knee. Previous studies reported that hardware is removed in approximately half of DIS patients due to local discomfort [20, 23, 24].

Secondary outcome measures of this study were antero-posterior knee laxity and functional outcome scores. At follow-up, the median side-to-side difference in antero-posterior translation measured with the Rolimeter® was 2 mm. In general, side-to-side difference in AP laxity is widely used to measure the success of ACL reconstruction and a delta of > 2 mm is defined as failure [26, 29, 30].

Data published by other groups showed similar results: in the pilot study of 10 patients by Eggli et al. [31] a median anterior translation difference after 24 months of 1 mm and of 2 mm after 60 months of 2 mm was reported.

In the case series by Kohl et al. [23], the antero-posterior translation differed from the normal knee by a mean of 1.2 mm at 24 months. In the cohort of Büchler et al. [24], the mean difference in antero-posterior translation between operated and healthy limb assessed with a KT-1000 was 0.0 ± 1.6 mm at 12 months of follow-up.

Henle et al. [20] published a mean anterior translation difference between the injured and the healthy contralateral knee after 3 months of 0.8 mm and after 24 months of 2.3 mm. The Lachman test of 26 patients in the study of Kösters et al. [32] showed a mean anterior translation difference to the healthy side of 1.7 mm after 12 months.

The reasons for such discrepancies are unknown, but they may be due to different combinations of injuries, to different mechanisms of compensation and to differences in rehabilitation. AP-translation measurements after surgery are only a surrogate measure for the re-established static stability of the knee joint, whereas proprioception is a key aspect in measuring the overall outcome of an ACL reconstruction [33]. The proportions and magnitudes of these proprioceptive contributions by the anterior cruciate ligament are currently under intense scientific investigation.

The patients in the present study reported a satisfaction of 9 on VAS after 1 year [22]. Clinically, our patients exhibited a practically normal knee function after 1 year with a median Lysholm score of 95, an IKDC score of almost 90 and with the same Tegner scale as before the ACL rupture.

Henle et al. showed after three years, a mean Lysholm, IKDC and Tegner scores of 96.2, 93.6.

A meta-analysis of Biau et al. [34] reported that only about 40% of patients made a full recovery after ACL reconstruction, with only 33% having a normal IKDC score after a semitendinosus transplant and 41% having a normal IKDC after a BTB (ligamentum patellae) transplant. They concluded that more than 60% did not fully recover (final overall IKDC score class A) after reconstruction.

The present patients obtained good functional scores and high levels of satisfaction. The successfully treated patients had a good clinical outcome based on antero-posterior knee laxity and the clinical scores were comparable to patients treated by autograft reconstruction. Still, the question if preservation of the remnants of the native ACL even without complete healing may play a role in regaining proprioceptive sense must be raised. It has yet to be investigated with further studies whether DIS can maintain its good results over a long-term follow-up or will be associated with even increased failure rate over time.

According to this study, DIS was more successful than conservative treatment, but less so than reconstruction. The findings of this study underline the inferiority of surgical outcome after DIS in young patients with failure rates reaching 16.3%, which is much higher than reported before.

Long-term studies are necessary to identify the ideal candidate for this procedure and further refinement. Until that time, DIS was not the preferred alternative to primary ACL reconstruction with autografts.

The strength of this study was to recall data from four, developer independent, centres in order to improve statistical interpretation. Being a case series, the present study generates level four evidence.

The small sample size, the retrospective character and the lack of a control group are the major drawbacks of the presented analysis. In addition, 12% of the study population was lost to follow-up and the follow-up is not long enough to address post-traumatic arthritis.

It must be recognized that there are a considerable number of potential factors not controlled in this analysis that may have a potential influence on the outcomes, such as different surgeon's experience, different rehabilitation protocols, different populations. The motivation of using this promising novel technique and the need to do surgery within a 21 days’ timeframe may have led to less rigorous indications and selection bias.

The primary intention of this repair-based approach is to maintain joint structures around the knee joint and limit further need for graft harvesting. The authors also want to point out another technical disadvantage of this surgical technique apart from the bulky tibial implant and that refers to the polyethylene wire that is left within the knee joint cavity of the patients. The outcome of such implant is not well established over time and needs further investigations.

## Conclusion

The optimal way of treating ACL ruptures is still under debate. With a residual antero-posterior translation under 3 mm, DIS restores in the majority of patients functional, but not necessarily normal, stability. Nevertheless, the relatively high re-operation rate is questioning the self-healing capacity of the torn ligament and better preoperative patient characterization is needed to better predict which patients benefit most from this new technique of preservation.

The present study considers DIS as an additional treatment option for acute ACL-rupture, but not dedicated for young age (< 25) or high level of sport activity population.

The optimal patient population to address for this surgery remains unclear. However, middle-aged patients with a moderate pre-injury sports activity level may benefit from this procedure as long as the MRI studies confirm a femoral footprint avulsion of the ACL.
